# The Latent Structure of Depression and Anxiety Symptoms: A Bifactor Exploratory Structural Equation Model of the PHQ‐9 and GAD‐7 in Primary Care

**DOI:** 10.1002/mpr.70089

**Published:** 2026-06-11

**Authors:** Gabriel Esteller‐Collado, Maider Prieto‐Vila, Celia Antuña‐Camblor, María Carpallo‐González, César González‐Blanch, Paloma Ruíz‐Rodríguez, Juan Antonio Moriana, Leonardo Adrián Medrano, Antonio Cano‐Vindel, Roger Muñoz‐Navarro

**Affiliations:** ^1^ Department of Personality Assessment and Psychological Treatments, Faculty of Psychology University of Valencia Valencia Spain; ^2^ Faculty of Psychology and Health Science Madrid Open University (UDIMA) Madrid Spain; ^3^ Department of Psychology International University of La Rioja (UNIR) Logroño Spain; ^4^ Mental Health Centre Marqués de Valdecilla University Hospital (IDIVAL) Santander Spain; ^5^ Tres Cantos Primary Care Centre Health Service of Madrid Madrid Spain; ^6^ Department of Psychology Faculty of Psychology University of Cordoba Cordoba Spain; ^7^ Maimonides Biomedical Research Institute of Cordoba (IMBIC) Cordoba Spain; ^8^ Reina Sofía University Hospital Cordoba Spain; ^9^ Department of Psychology Pontificia Universidad Católica Madre y Maestra Santiago de los Caballeros Dominican Republic; ^10^ Department of Experimental Psychology Faculty of Psychology Cognitive Processes and Speech Therapy Complutense University of Madrid Madrid Spain

**Keywords:** anxiety, bifactor model, depression, exploratory structural equation modelling, primary care, transdiagnostic

## Abstract

**Introduction:**

The high comorbidity between depression and anxiety challenges traditional nosological models. In order to better reflect this overlap, dimensional approaches seek to clarify whether these symptoms reflect a single underlying construct of general distress or a more complex multidimensionality. The aim was to examine the underlying structure of depression and anxiety symptoms, measured by the PHQ‐9 and GAD‐7, by estimating and comparing three factor models: one‐factor, two‐factor correlated, and bifactor.

**Methods:**

Data from 1704 primary care (PC) patients from the PsicAP clinical trial were analysed. Dimensionality was assessed using hierarchical Exploratory Graph Analysis (EGA), and models were estimated using Exploratory Structural Equation Modelling (ESEM). Model fit was compared using the *χ* ^2^, CFI, TLI, RMSEA, and SRMR indices.

**Results:**

The bifactor model offered the most acceptable comparative fit to the data (CFI = 0.956; TLI = 0.928; RMSEA = 0.076; SRMR = 0.028). Bifactor indices revealed a relevant but not dominant general factor (ECV = 0.40), indicating it does not account for sufficient variance to justify an essentially unidimensional interpretation. Furthermore, the specific factors of depression and anxiety emerged as well‐defined constructs (*H* > 0.75) with modest reliable specific variance (ωHS: 0.22 and 0.27, respectively).

**Discussion:**

The findings suggest a hierarchical structure where a general factor of distress coexists with specific factors. This suggests the potential utility of considering a multi‐level perspective in clinical assessment, accounting for both shared distress and specific symptom profiles.

## 1 Introduction

Depression and anxiety disorders are among the most prevalent and disabling mental health problems globally (World Health Organization 2022, 2025). Their high prevalence is associated with a substantial deterioration in quality of life, a significant reduction in psychosocial functioning, and a considerable burden on public health systems (GBD 2022; Rief et al. 2024).

Clinically, one of the most relevant characteristics between depression and anxiety is their high comorbidity (Jacobson and Newman 2017; Kessler et al. 2015). The frequent co‐occurrence of both symptom sets challenges the validity of traditional nosological classification systems, such as the DSM‐5 or the ICD‐11 (Cuthbert 2014; Insel et al. 2010). The considerable overlap in diagnostic criteria, as well as the symptomatic heterogeneity within each category, has prompted the search for dimensional models that more reliably capture the latent structure of emotional psychopathology (Caspi et al. 2014; Caspi and Moffitt 2018; Kotov et al. 2021).

In this context, transdiagnostic approaches have emerged in recent decades as a comprehensive and parsimonious theoretical framework for explaining this comorbidity (Abramovitch et al. 2021; Dalgleish et al. 2020; Sauer‐Zavala et al. 2017). This perspective posits that, beyond specific symptomatic manifestations, there are common aetiopathogenic and maintenance processes among different disorders (Ciharova et al. 2021; Muñoz‐Navarro et al. 2025; Sloan et al. 2017). Furthermore, one of the cornerstones of this approach is the existence of a general factor (GF) of emotional distress that underlies the shared symptomatology between depression and anxiety (Barlow et al. 2014; Clark and Watson 1991; Watson and Naragon‐Gainey 2014).

The empirical study of the latent structure of these disorders has traditionally been approached by analysing the factor structure of the measurement instruments (Clark and Watson 2019). Previous literature has frequently compared a one‐factor model, which conceptualises depression and anxiety as manifestations of a single construct of general distress, with a two‐factor correlated model, which posits the existence of two distinct but related constructs (Annunziata et al. 2011; Bocéréan and Dupret 2014; Norton et al. 2013). However, these models fail to adequately capture the complexity of the relationship between the common and specific variance of each construct (Jovanović et al. 2021; Norton et al. 2013; Yılmaz Koğar and Koğar 2023). A more sophisticated theoretical and methodological alternative is the bifactor model (Reise 2012; Rodriguez et al. 2016), which allows the variance of items to be simultaneously decomposed into a GF and multiple specific factors. The bifactor model is therefore particularly consistent with the transdiagnostic framework, as it explicitly models both the common and distinctive elements of psychopathology, offering a more nuanced representation of its latent structure (Caspi et al. 2014; Lahey et al. 2012).

Much of the previous research on this issue has used Confirmatory Factor Analysis (CFA) (Norton et al. 2013; Stochl et al. 2022; Yeung et al. 2020). However, CFA imposes the restriction of setting the factor loadings of items in non‐main factors to zero (Brown 2015). This limitation, which is often unrealistic, is particularly problematic when analysing highly interrelated constructs such as depression and anxiety, as it can lead to poor model fit and an overestimation of factor correlations (Marsh et al. 2014; Morin et al. 2020). Exploratory Structural Equation Modelling (ESEM) emerges as an alternative that integrates the flexibility of exploratory factor analysis into a confirmatory framework, allowing for the free estimation of cross‐loadings and potentially offering a more accurate representation of the data structure (Marsh et al. 2020).

The aim of this study is to examine the underlying factor structure of symptoms of depression and anxiety, measured by the PHQ‐9 and the GAD‐7. Specifically, we combine hierarchical Exploratory Graph Analysis (EGA) to assess initial dimensionality with the flexibility of the ESEM approach. To this end, we estimated and compared the fit of three theoretical models in a large clinical sample of primary care (PC) patients: a one‐factor model of general distress, a two‐factor correlated model, and a bifactor model. This analysis seeks to clarify whether a GF of distress predominates in this healthcare setting strongly enough to justify unidimensional approaches, or whether the structure instead reflects complex multidimensionality, requiring the consideration of both shared and specific variance in each disorder.

## 2 Method

### 2.1 Participants and Procedure

The data for this study come from the PsicAP project (Cano‐Vindel et al. 2016), a multicentre, two‐arm, single‐blind randomised clinical trial (RCT) conducted in Spain. This RCT has demonstrated the efficacy of group‐based TD‐CBT for the treatment of emotional disorders in PC (Cano‐Vindel et al. 2021). For this study, data from the pre‐treatment assessment of all patients who were evaluated prior to their potential inclusion in the RCT and signed the informed consent form (*N* = 1704) were used. Therefore, this sample consists of patients referred by their general practitioners on suspicion of depression and/or anxiety disorders who agreed to undergo a psychological assessment, which is consistent with a sample of PC patients seeking treatment. These patients completed a battery of computerised self‐report questionnaires under the supervision of a research psychologist.

### 2.2 Instruments

*Patient Health Questionnaire‐9 (PHQ‐9)*: The PHQ‐9 was used to assess depressive symptoms (Kroenke et al. 2001). This scale consists of nine items based on the DSM‐IV criteria for Major Depressive Disorder. The response format is a 4‐point Likert‐type scale (0 = never; 3 = almost every day). This questionnaire was validated in a sample of PC patients in Spain (Muñoz‐Navarro, Cano‐Vindel, Medrano, et al. 2017) and the internal consistency in the sample was good (*α* = 0.86).

*Generalized Anxiety Disorder‐7 (GAD‐7)*: The GAD‐7 was used to assess generalized anxiety symptoms (Spitzer et al. 2006). The questionnaire consists of 7 items with a 4‐point Likert‐type response scale (0 = never; 3 = almost every day). The patient is asked to respond according to the frequency with which he/she has experienced a range of symptoms in the last two weeks and the maximum score is 21 points. The Spanish version validated by Garcia‐Campayo et al. (2010), which has also been validated for the PC setting (Muñoz‐Navarro, Cano‐Vindel, Moriana, et al. 2017), was used. The internal consistency of the instrument in the sample was good (*α* = 0.87).

### 2.3 Statistical Analyses

Data management and analysis were conducted using the IBM SPSS statistical software program (v.29) and R (4.3.3).

First, the hierarchical latent dimensionality structure underlying the items of both scales was evaluated. To do this, hierarchical EGA was used. Hierarchical EGA was estimated using the graphical operator of contraction and absolute minimum selection (GLASSO) with the walktrap clustering algorithm (Brusco et al. 2022; Golino and Christensen 2022). Likewise, to determine whether a correlated constructs model or a bifactor model better represented the data, the generalised total entropy fit index (Generalised TEFI) was obtained for the lower‐ and higher‐order structures (Golino et al. 2024). Lower values in the generalised TEFI indicate a better fit. Both hierarchical EGA and generalised TEFI were calculated using the EGAnet (v.2.3.0) package in R, using the hierEGA and GenTEFI functions contained in that package (Golino and Christensen 2022).

Second, the factor structure was examined using exploratory structural equation modelling (ESEM) with the lavaan package in R (0.6–19). Three factor models were calculated and compared: a one‐factor model (ESEM‐1F), a two‐factor correlated model (ESEM‐2F), and a bifactor model (BI‐ESEM‐2F). For the ESEM‐1F and BI‐ESEM‐2F models, the factors were rotated using target rotation (Browne 2001). Moreover, for BI‐ESEM‐2F the target rotation was specified to be orthogonal to ensure identification and interpretation (Morin et al. 2020). Given that the items were ordinal‐categorical, the weighted least squares estimator with mean‐ and variance‐adjusted standard errors (WLSMV) was used (Rhemtulla et al. 2012). The factor loadings for the ESEM‐1F and ESEM‐2F models were considered low (0.40), medium (0.55) and high (0.70) (Garrido et al. 2013). For the bifactor solution, given that the variance is divided, the factor loadings were interpreted as low (0.30), medium (0.45) and high (0.60) (Garcia‐Garzon et al. 2019).

The fit of all models was evaluated using the comparative fit index (CFI), Tucker‐Lewis index (TLI), root mean square error of approximation (RMSEA), and standardised root mean square residual (SRMR) (Kline 2016; Marsh and Hocevar 1985). Robust versions of these indices were used. Values of CFI/TLI ≥ 0.90 and RMSEA/SRMR < 0.08 are considered indicative of an adequate fit. Higher CFI/TLI values and lower RMSEA/SRMR values are indicative of a better fit.

Finally, to better evaluate the quality of the bifactor solution, we calculated the explained common variance (ECV), omega (*ω*), hierarchical omega (ωH), hierarchical omega subscale (ωHS) and construct replicability index (H) using the BifactorIndicesCalculator (v.0.2.2) package in R (Dueber and Toland 2023). For the interpretation of these indices (Arias et al. 2018; Flores‐Kanter et al. 2018; Rodriguez et al. 2016), an ECV of the GF greater than 0.70 indicates that the structure is sufficiently unidimensional, and an ωH > 0.80 suggests that the total score is a reliable measure of a unitary construct. As for specific factors, their scores are considered interpretable if their corresponding ωHS is > 0.50. Finally, values ≥ 0.70 in the H index is indicative of a well‐defined latent variable.

## 3 Results

### 3.1 Sample Description

The sample had a mean age of 43.6 years (SD = 12.3) and was predominantly female (78.6%). Most were married (46.4%), worked part‐time (37.5%) and had an annual income of 24,000€ or less (80.4%). In terms of educational level, approximately half of the sample (47.2%) had completed an intermediate level of education (Table 1).

**TABLE 1:** Demographics characteristics of sample.

Characteristics	Total (*n* = 1704)	
	*n*	%
Gender		
Female	1340	(78.6)
Male	364	(21.4)
Age group, years		
≤ 19	27	(1.6)
20–39	612	(35.9)
40–59	910	(53.4)
≥ 60	155	(9.1)
Marital status		
Married	790	(46.4)
Divorced	154	(9.0)
Widowed	55	(3.2)
Separated	87	(5.1)
Never married	358	(21.0)
Unmarried	260	(15.3)
Level of education		
No schooling	25	(1.5)
Basic education	432	(25.4)
Secondary education	371	(21.8)
High school	434	(25.4)
Bachelor	366	(21.5)
Master/doctorate	76	(4.5)
Employment situation		
Employed full‐time	250	(14.7)
Employed part‐time	639	(37.5)
Unemployed, in search of work	367	(21.5)
Unemployed, not looking for work	205	(12.0)
Temporary incapacity to work	129	(7.6)
Permanent incapacity to work	38	(2.2)
Retired	76	(4.5)
Level of income (per year)		
Less than 12,000€	673	(39.5)
Between 12,0000€–24,000€	697	(40.9)
Between 24,0000€–36,000€	221	(13.0)
More than 36,000€	113	(6.6)

*Note:* Age group expressed in years.

Regarding the study variables, the mean score for depression (PHQ‐9) was 13.3 (SD = 6.4) and for anxiety (GAD‐7) 11.7 (SD = 5.2). The correlation between the two constructs was high (*r* = 0.705; *p* < 0.001).

### 3.2 Dimensionality Assessment

**FIGURE 1:**
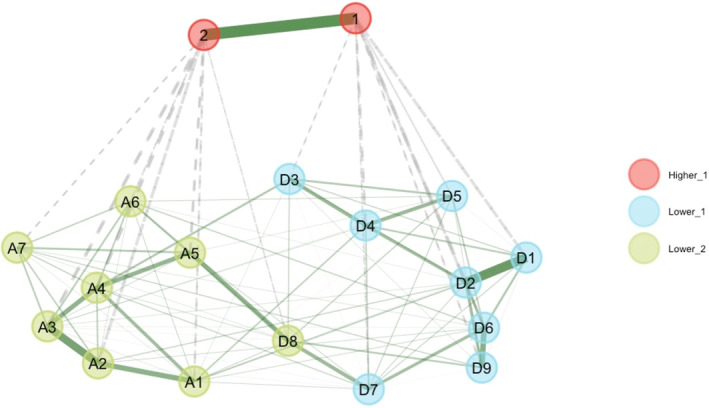
Dimensionality assessment with hierarchical exploratory graph analysis. A1–A7: anxiety items; D1–D9: depresson items.

The dimensionality assessment with hierarchical EGA suggested a two‐level latent structure: the lower level composed of two factors and the upper level composed of a single latent variable (Figure 1). A notable finding in the lower‐order structure was that, in contrast to theory, item 8 of the depression scale (‘Moving or speaking so slowly that others may have noticed. Or, conversely, being so restless or agitated that you have been moving more than usual’) was empirically grouped with the community of anxiety items. In addition, the Generalised TEFI index yielded a fit value of −10.5 for the lower‐order structure and −13.8 for the higher‐order structure. The lower value for the higher‐order structure suggests that the bifactor model may fit the data better than the correlated constructs model.

### 3.3 Factor Analyses

**FIGURE 2:**
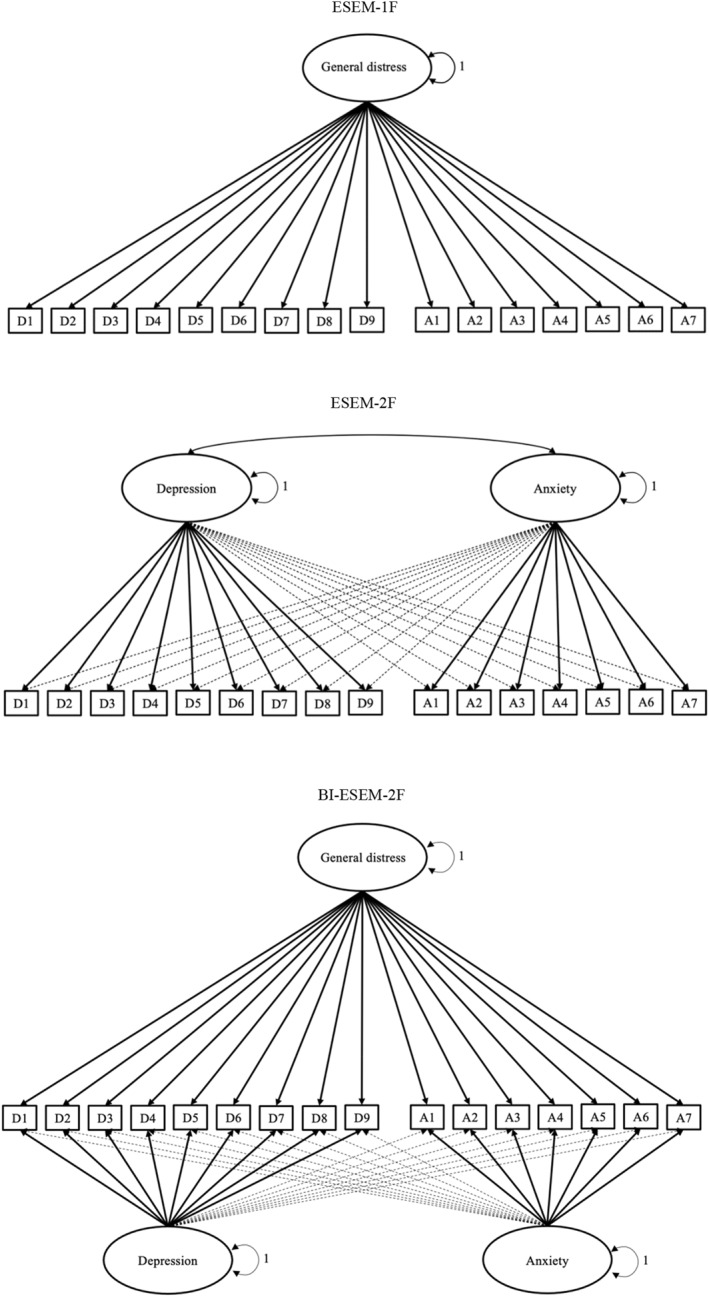
Factor models of the study. Squares represent observed variables. Ovals represent latent variables. Black unidirectional arrows linking ovals and rectangles represent the target factor loadings. Discontinuous unidirectional arrows linking ovals and rectangles represent the cross‐loadings. Bidirectional arrows connecting a single oval represent factor variances. Bidirectional arrows connecting two ovals represent the factor covariances.

Following the results obtained in the hierarchical EGA, three factor models were estimated and compared to explain the underlying structure of the data (Figure 2). First, a one‐factor model with a single underlying latent factor of general distress (ESEM‐1F) was estimated. This model showed an inadequate fit in the indicators, suggesting a poor correspondence with the observed data (Table 2). Second, a two‐factor correlated model (ESEM‐2F) was estimated. In this case, the model presented acceptable fit indicators for CFI, TLI, and SRMR, remaining within the desirable ranges (Table 2). However, the RMSEA values exceeded the threshold of 0.08. Finally, a bifactor model (BI‐ESEM‐2F) was estimated, which included two specific factors and one GF coexisting. This model shows the most acceptable comparative fit relative to the previous ones, with CFI values above 0.95, TLI values above 0.90, and RMSEA and SRMR values below 0.08.

**TABLE 2:** Fit indices for the factorial models.

Model	*χ* ^2^	df	CFI	TLI	RMSEA	SRMR
ESEM‐1F	1753.4***	104	0.810	0.781	0.133	0.069
ESEM‐2F	488.4***	89	0.921	0.894	0.093	0.039
BI‐ESEM‐2F	233.8***	73	0.956	0.928	0.076	0.028

*Note:* The robust indices are reported in the CFI, TLI, and RMSEA indices.

Abbreviations: *χ* ^2^ = chi‐square; CFI = comparative fit index; df = degrees of freedom; SRMR = standardised root mean square residual; RMSEA = root mean square error of approximation; TLI = Tucker‐Lewis index.

*p < 0.05.

**p < 0.01.

^***^ *p* < 0.001.

The factor loadings and factor correlations for all models are shown in Table 3. The average principal loadings in the ESEM‐2F model were medium for depression (0.54) and high for anxiety (0.71). In the case of depression, the average cross‐factor loadings were low (0.24), with some particularly noteworthy ones, such as item D8 (0.51), which loaded more strongly on the anxiety factor than on the depression factor (as suggested by the hierarchical EGA). In the case of anxiety, the average cross‐loadings were low (0.06), with only one item (A1) showing a particularly increased cross‐loading (0.21). Analysis of the factor loadings of the ESEM‐2F model suggests that both factors have asymmetric specificity. While anxiety is a relatively well‐defined construct, the depression factor shows considerable overlap, evidenced by the magnitude of its cross‐loadings.

**TABLE 3:** Standardised factor loadings and correlations for the factorial models.

Item/factor	ESEM 1‐F		ESEM‐2F			BI‐ESEM‐4F			
	GF	*h* ^*2*^	F1	F2	*h* ^*2*^	F1	F2	GF	*h* ^*2*^
D1	0.79	0.63	0.86	0.02	0.76	0.69	−0.16	0.60	0.77
D2	0.86	0.73	0.83	0.13	0.83	0.68	−0.08	0.65	0.85
D3	0.60	0.37	0.43	0.27	0.39	0.44	0.18	0.32	0.40
D4	0.70	0.48	0.57	0.23	0.53	0.55	0.10	0.42	0.53
D5	0.58	0.34	0.40	0.27	0.36	0.46	0.20	0.25	0.39
D6	0.73	0.53	0.57	0.27	0.57	0.55	0.13	0.44	0.58
D7	0.68	0.46	0.42	0.36	0.48	0.48	0.27	0.32	0.51
D8	0.65	0.42	0.22	0.51	0.44	0.37	0.48	0.17	0.55
D9	0.63	0.40	0.56	0.17	0.46	0.57	0.09	0.32	0.48
A1	0.77	0.60	0.21	0.65	0.62	0.21	0.41	0.59	0.63
A2	0.81	0.66	0.03	0.83	0.72	−0.02	0.49	0.76	0.82
A3	0.82	0.67	−0.01	0.87	0.76	0.02	0.56	0.65	0.76
A4	0.78	0.61	0.05	0.79	0.67	0.12	0.62	0.48	0.69
A5	0.64	0.41	0.02	0.68	0.47	0.17	0.65	0.19	0.58
A6	0.59	0.35	0.08	0.57	0.39	0.14	0.45	0.35	0.39
A7	0.56	0.32	0.03	0.58	0.36	0.09	0.45	0.34	0.36
F1			1			1			
F2			0.57	1		0.00	1		
GF						0.00	0.00	1	

*Note:* In the ESEM‐1F and ESEM‐2F models, factor loadings ≥ 0.40 in absolute value are highlighted in grey (Garrido et al. 2013). In the BI‐ESEM‐2F model, factor loadings ≥ 0.30 in absolute value are highlighted in grey (Garcia‐Garzon et al. 2019).

Abbreviations: A1–A7 = anxiety items; D1–D9 = depression items; ESEM = exploratory structural equation modelling; *h* ^2^ = communality.

In the BI‐ESEM‐2F model, the average factor loadings in the GF were medium (0.42), with all items loading significantly in the GF except for items D5, D8, and A5 (D5: appetite; D8: mobility; A5: difficulty relaxing). Regarding specific factors, the average factor loadings were considered medium for depression (0.53) and anxiety (0.51). Once again, item D8 for depression showed a higher loading on the anxiety construct than on the depression construct, although to a lesser extent than in the ESEM‐2F model. The average cross‐factor loadings for depression and anxiety were low, 0.18 and 0.11 respectively. Analysis of the factor loadings of the BI‐ESEM‐2F model suggests that the bifactor model offers a more adequate representation of the latent structure. The inclusion of the GF manages to isolate the common variance and allows the specific factors of depression and anxiety to emerge more clearly and distinctly. Likewise, the attenuation of cross‐loadings, including in the case of item D8, supports the idea that the high overlap observed in the ESEM‐2F model could be an artefact derived from not explicitly modelling the common variance through the GF.

Finally, we examined the complementary indices obtained for the BI‐ESEM‐2F model. The GF produced an ECV = 0.40, indicating that the specific factors contributed a majority proportion of the common variance (ECV = 0.60). The ECVs for the specific factors were.28 for depression and 0.32 for anxiety. The *ω* for the total scores was 0.91, indicating high reliability. However, the ωH value for the GF was 0.41. This result shows that the GF explained only 45% of the reliable variance (0.41/.91), discouraging an essentially unidimensional interpretation. To evaluate the specific dimensions, we differentiated between construct replicability and score interpretability. The H index indicated that the GF (0.85) and the specific factors of Depression (0.77) and Anxiety (0.81) were well‐defined and replicable latent constructs (all > 0.70). Conversely, the ωHS values for the specific factors were low (0.22 for Depression and 0.27 for Anxiety). This indicates that while the specific latent constructs are well‐defined, the corresponding subscale scores retain limited unique reliable variance once the GF is accounted for. Overall, these indices support a multidimensional structure but caution against interpreting the specific subscale scores as independent measures.

## 4 Discussion

The aim of this study was to examine the factor structure underlying depression and anxiety symptoms, measured using the PHQ‐9 and GAD‐7, in a large sample of PC patients. To do so, three factor models were estimated and compared using ESEM methodology: one‐factor, two‐factor correlated, and bifactor. The results showed that the bifactor model (BI‐ESEM‐2F) offered the most acceptable comparative fit to the data among the evaluated models, yielding adequate fit indices. These findings support a multidimensional latent structure of PHQ‐9 and GAD‐7 composed of a GF of distress and two specific factors of depression and anxiety.

The bifactor model is consistent with a large body of literature that suggests this structure as a theoretically plausible representation of the high interrelationship between depression and anxiety symptoms (Carmichael et al. 2023; Iani et al. 2014; Jovanović et al. 2021; Norton et al. 2013; Sun et al. 2022; Teymoori et al. 2020; Yılmaz Koğar and Koğar 2023) while also offering empirical consistency with the foundations of the transdiagnostic approach. Theoretically, the high comorbidity among emotional disorders has been explained by the existence of a general vulnerability factor, often conceptualised as negative affectivity or neuroticism (Barlow et al. 2014) or as a general psychopathology ‘*p’* factor (Caspi et al. 2014; Caspi and Moffitt 2018). The GF of distress identified in our model can tentatively be interpreted as an empirical indicator of this underlying transdiagnostic construct, although this is merely a proposal based on structural fit. Furthermore, our findings reveal not only a GF of distress, but also the simultaneous coexistence of two specific factors of depression and anxiety. While our study is inherently limited to the symptoms captured by two specific scales, these results align with the logic of hierarchical models of psychopathology, such as the HiTOP model (Kotov et al. 2021), which propose that psychopathology is structured across multiple levels, with broad spectrums and more specific subfactors.

The use of the ESEM methodological framework, which allows the estimation of cross‐loadings, was key to properly testing these structures against traditional CFA models, which impose often unrealistic zero‐loading restrictions (Brown 2015; Marsh et al. 2014). However, our results show that the flexibility of ESEM alone is not enough. For example, the ESEM‐2F model revealed substantial and theoretically problematic cross‐loadings, pointing to poor discriminant validity. The BI‐ESEM‐2F model specification addressed this issue more adequately by modelling common variance through the GF, resulting in an attenuation of cross‐loadings. These results suggest that the problematic covariance between factors was, at least in part, an artefact of unmodelled shared general variance. Therefore, the combination of the flexibility of ESEM with the theoretical specification of a bifactor model (Morin et al. 2020) provided an improved descriptive fit and offered a pragmatic approach to decomposing the variance, although this descriptive advantage should not be taken as a decisive proof of a definitive structural resolution.

Another key aspect of our findings is the specific distribution of variance revealed by the bifactor indices. As indicated by the ECV (0.40) and the ωH (0.41), the GF does not account for sufficient reliable variance to support a strong GF or an essentially unidimensional interpretation of the scales (Rodriguez et al. 2016). Concurrently, the specific factors also exhibited low ωHS values (0.22 for depression and 0.27 for anxiety), indicating that the reliable specific variance is modest once the GF is accounted for. Therefore, our results highlight an important distinction between construct replicability and score interpretability. While the high H values (> 0.75) suggest that the specific depression and anxiety constructs captured by the PHQ‐9 and GAD‐7 are well‐defined and likely to be replicable within similar PC populations, the low ωHS values (< 0.30) indicate that their corresponding subscale scores retain limited unique reliable variance once the GF is accounted for. Consequently, rather than supporting the use of these subscales as independent measures, our findings are consistent with a complex multidimensionality where general and specific variances are inextricably linked. This finding is noteworthy, as it qualifies the results of other studies which, while supporting the bifactor structure, have reported a much more dominant GF (Carmichael et al. 2023; Iani et al. 2014; Teymoori et al. 2020), or recent large‐scale studies that have strongly supported the use of the sum of the PHQ‐9 and GAD‐7 total scores, based on a GF that explains up to 80% of the variance (Stochl et al. 2022). One possible explanation for this discrepancy lies in the context of the study. While Stochl et al. (2022) analysed a sample receiving specialised psychotherapy, our PC sample is characterised by high heterogeneity and significant symptomatic overlap, often with a strong presentation of somatic symptoms (Löwe et al. 2008). It is plausible that in this ‘front‐line’ context, specific variance (e.g., the anhedonia of depression vs. the worry and tension characteristics of anxiety) is particularly pronounced and clinically distinctive, in contrast to more homogeneous general population or psychiatric samples. In this setting, using an aggregate score from the PHQ‐9 and GAD‐7 as a single measure of general distress would be inadequate, just as interpreting the specific subscales in complete isolation would be misleading. Clinical assessment should take into account both shared and specific variances.

Finally, another finding of notable interest was the anomalous performance of item D8 of the PHQ‐9 (‘moving or talking so slowly… or, conversely, being so restless or agitated…’). In both the EGA analysis and the ESEM factorial models, this item showed a strong association with the anxiety construct. This result is consistent with previous research that has pointed to the factor complexity of the somatic and psychomotor symptoms of depression (Boothroyd et al. 2019; Fried 2017). The dual nature of this item, which assesses both psychomotor retardation and agitation, introduces a potential source of bias into the item. From a psychometric perspective, this combined wording could pose a measurement problem, as it is plausible that the agitation component shares variance with the physiological arousal and restlessness characteristic of anxiety, which are explicitly measured in the GAD‐7 (e.g., ‘Being so restless that it is difficult to sit still’). Consequently, the cross‐loadings observed for item D8 may be partly due to a methodological artefact related to the wording of the item, rather than reflecting exclusively a substantial clinical overlap between the latent constructs of depression and anxiety as operationalised by these specific instruments. This observation warrants a cautious interpretation of item D8 as an unambiguous indicator of depression and underscores the importance of precise symptom formulation in clinical assessment.

### 4.1 Clinical Implications

The findings of this study suggest potential theoretical implications for clinical assessment practice using these instruments, especially in PC. While our cross‐sectional psychometric design precludes testing clinical outcomes, the evidence of a multidimensional structure theoretically aligns with a multilevel assessment and treatment approach. On the one hand, the existence of a shared GF of distress captured by these scales is consistent with the rationale underlying transdiagnostic approaches, such as the Unified Protocol (Barlow et al. 2017) or the PsicAP protocol (Cano‐Vindel et al. 2021), which focus on common underlying mechanisms (e.g., emotional regulation, behavioural activation, or cognitive restructuring). Conversely, the presence of significant specific factors indicates that clinicians should not ignore particular symptom profiles when interpreting PHQ‐9 and GAD‐7 scores. Consequently, we hypothesize that an integrative assessment and therapeutic approach might theoretically benefit from beginning with transdiagnostic modules to address general distress, followed by specific components according to the patient's symptomatic profile. Furthermore, based solely on our structural findings, we speculate that relying exclusively on a combined score from both questionnaires as the sole follow‐up measure might mask clinically relevant variations, and that monitoring both general and specific symptom trajectories using these specific tools could be more informative.

### 4.2 Limitations

This study has several limitations that should be acknowledged. First, the assessment of symptoms was based exclusively on self‐report measures. Although these instruments are widely used and have been validated in the specific context of the study sample (Muñoz‐Navarro, Cano‐Vindel, Medrano, et al. 2017; Muñoz‐Navarro, Cano‐Vindel, Moriana, et al. 2017), they are susceptible to response biases, such as social desirability. Second, although the sample is large and clinically representative of the PC context in Spain, its generalisation to other populations should be done with caution. PC patients often present high somatic comorbidity and mild to moderate levels of distress, so this factor structure may not be identical in community (non‐clinical) samples or in samples of psychiatric patients with more severe disorders. Related to this, the sample was predominantly female (78.6%), which, although representative of the population seeking psychological care at this level of care, limits the generalisation of the findings to men. Thirdly, the findings are specific to the instruments used, and the factor structure could vary when using other measures that cover a different symptomatic spectrum. Therefore, the findings should be interpreted specifically as the structural organization of the symptoms captured by the PHQ‐9 and GAD‐7, rather than a definitive representation of the entire anxiety and depression constructs. However, other studies have found similar structures with other inventories, such as the HADS (Carmichael et al. 2023) or the DASS‐21 (Yılmaz Koğar and Koğar 2023). Fourthly, it is necessary to acknowledge a methodological limitation inherent in bifactor models. As noted in the psychometric literature, bifactor models tend to yield better fit indices because they estimate more parameters than standard correlated‐factors models (Rodriguez et al. 2016). Despite this statistical advantage, the choice of the bifactor model in this study was also driven by empirical and theoretical considerations. Empirically, the ESEM‐2F model exhibited higher cross‐loadings than the BI‐ESEM‐2F, which could indicate lower discriminant validity between factors. The bifactor specification mitigates (although it does not completely eliminate) this problem by explicitly modelling the shared variance through the GF. Theoretically, this structure is consistent with the transdiagnostic conceptualisation of emotional disorders. Therefore, whilst we acknowledge the statistical tendency of bifactor models to fit better than correlated‐factor solutions, our interpretation is based not only on model fit, but also on complementary bifactor indices and theory.

### 4.3 Future Lines and Conclusion

The limitations mentioned above open up lines for future research. It would be a priority to carry out measurement invariance analyses to confirm whether this bifactor structure is equivalent between men and women, given the female predominance in the sample. It is also necessary to replicate these findings in diverse clinical and cultural samples to establish the generalisability of the structure. Beyond replication, it would be interesting to examine the differential predictive validity of factor scores (general and specific) on clinical outcome variables, such as response to transdiagnostic treatment modules versus specific modules. Finally, the incorporation of other methodological frameworks of analysis, such as network analysis, could offer a complementary view, allowing us to explore the direct symptom‐to‐symptom interactions that underlie the covariance modelled by our latent factors.

In conclusion, this study suggests, through the combination of ESEM methodology and bifactor modelling, that the structure of the symptoms measured by the PHQ‐9 and GAD‐7 in PC can be characterized by a dual nature. Far from being simply one‐dimensional, the findings reveal that the variance of these instruments is best explained by the coexistence of a GF of distress alongside specific factors of depression and anxiety. This nuance is clinically relevant, suggesting that comprehensive assessment and treatment planning in this setting could benefit from considering both the shared and specific dimensions captured by these instruments.

## Author Contributions

**Gabriel Esteller‐Collado:** conceptualization, investigation, methodology, formal analysis, visualization, writing – original draft, writing – review and editing, funding acquisition. **Maider Prieto‐Vila:** investigation, writing – review and editing. **Celia Antuña‐Camblor:** investigation, writing – review and editing. **María Carpallo‐González:** investigation, writing – review and editing. **César González‐Blanch:** investigation, writing – review and editing. **Paloma Ruíz‐Rodríguez:** investigation, writing – review and editing. **Juan Antonio Moriana:** investigation, funding acquisition, writing – review and editing. **Leonardo Adrián Medrano:** investigation, writing – review and editing. **Antonio Cano‐Vindel:** supervision, project administration, funding acquisition, investigation, writing – review and editing. **Roger Muñoz‐Navarro:** supervision, funding acquisition, investigation, writing – review and editing.

## Funding

This work is part of the projects funded by MICIU/AEI/10.13039/501100011033, through the projects: PID2019‐107243RB‐C21, PID2019‐107243RB‐C22 and CPP2023‐010817. It was also a project developed within the framework of the General Collaboration Agreement between the University of Valencia and Banco Santander, S.A., as part of the Santander Postdoctoral Research Grants. Finally, this project has also received support from the José Castillejo Grant (CAS2023/00397) awarded by the MCIN/AEI/10.13039/501100011033. These entities only provided funding for the recruitment of research staff and played no role in the design of the trial, data collection, analysis or writing of this manuscript.

## Ethics Statement

The study was approved by the National Scientific Research Ethics Committee in Spain, conducted in accordance with the Declaration of Helsinki (EUDRACT: 2013‐001955‐11) and the study protocol was registered (ISRCTN58437086).

## Consent

All participants gave their written informed consent.

## Conflicts of Interest

The authors declare no conflicts of interest.

## Data Availability

The data that support the findings of this study are available from the corresponding author upon reasonable request.
